# In Situ Coupling Applied Voltage and Synchrotron Radiation: Operando Characterization of Transistors

**DOI:** 10.1186/s11671-022-03662-y

**Published:** 2022-02-02

**Authors:** Anton Davydok, Yuriy N. Luponosov, Sergey A. Ponomarenko, Souren Grigorian

**Affiliations:** 1 Institute for Materials Physics, Helmholtz-Zentrum Hereon, Notkestr 85, 22607 Hamburg, Germany; 2 Enikolopov Institute of Synthetic Polymeric Materials of the Russian Academy of Sciences, Profsoyuznaya 70, Moscow, Russia; 3 Department of Physics, University of Siegen, Walter-Flex-Straße 3, 57072 Siegen, Germany; 4 Department of Chemistry, Sapienza University of Rome, p.le Aldo Moro 5, 00185 Rome, Italy

**Keywords:** OFETs, Operando studies, nanoGIWAXS, nanoGIXD, *α*,*ω*-dihexyl-*α*-quaterthiophene

## Abstract

A compact voltage application setup has been developed for in situ electrical testing of organic field effect transistors in combination with X-ray scattering studies at a synchrotron beamlines. Challenges faced during real condition in-operando test of newly developed OFETs originated an idea of creation of a new setup which excludes number of factors that make experiments complicated. The application of the setup is demonstrated on a prototype of an organic transistors based on *α*,*ω*-dihexyl-*α*-quaterthiophene molecules. The new setup allows to monitor material structural changes by X-ray scattering under applied voltage conditions and their direct correlations. The versatile setup eliminates possible shadowing effects and short circuits due to misalignment of the contacts. The electrical stability of the prototypes was characterized by the application of different voltage values. Corresponding structural changes were monitored by grazing X-ray scattering technique before, during and after the voltage was applied. The selected oligothiophene material with proved transistor properties shows high stability and directional anisotropy under applied voltage conditions. Thanks to a compact and flexible design of the setup, different type of small dimension devices could be studied under external voltage conditions at various synchrotron beamlines.

**Graphical Abstract:**

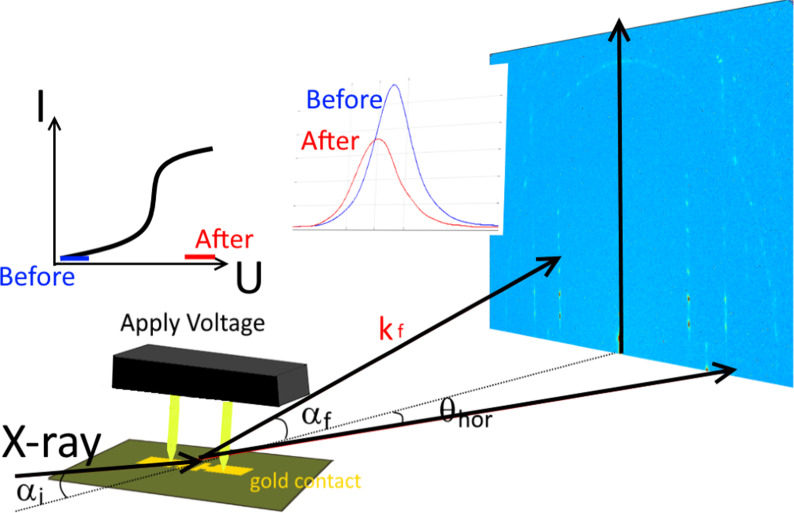

## Introduction

During last decades, organic conjugated material-based devices demonstrate huge potential for new generation of low-cost electronics. Recent reviews report on the semiconducting organic materials of different composition with outstanding electrical and optical properties for applications as functional materials in organic light emitting diodes [1], organic filed-effect transistors (OFETs) [2–4] or organic solar cells [5, 6].

Important role in the studies, before application in mass production, is playing a strong connection between investigated material properties and its microstructure and molecular ordering. Molecule with similar components but with different structure, ordering, distribution, and various bents might demonstrate very different properties and reaction to externally applied conditions [7, 8]. Rather recent studies shown relevance of the static and dynamic parameters in the electrical performances of OFETs and showed that the radiofrequency model established in a solid-state, it allows a reliable prediction of the performances of organic prototype. Authors are reporting on the effective miniaturization of OFETs, which is expected to provide a useful design guideline also in the integration of organic digital and analog circuits [9]. A lower limit of the mobility of ‘high-performance’ organic transistors has been established in the range of 1–10 cm^2^ V^−1^ s^−1^ [10]. However, this limit could be reached by rather short list of the organic materials such as single crystals or highly ordered films. Small molecules including oligothiophenes are promising candidates for organic electronics applications with mobilities up to 1 cm^2^ V^−1^ s^−1^ [11] and power conversion efficiency reaching of 10% [12]. Among different linear oligothiophenes, *α*,*α’*-dihexyl-quaterthiophene (DH4T) is well-known oligothiophenes revealing high field-effect mobility and well-ordered microstructure [13, 14]. In particular, a highly crystalline phase of DH4T with in-plane π-π orientation is beneficial for charge transport [15].

The unique properties of organic semiconductors, issues, and problems being non-typical for inorganic transistors are described in [13]. Important place in this research is taken by in situ experiments, in particular by in situ investigation using X-ray diffraction and scattering techniques [16–18]. Since most of the smart devices are designed as layered systems on substrate including electrodes and deposited organic material, an instrument sensitive to surfaces and interfaces is necessary. The technique of grazing X-ray scattering/diffraction with controllable penetration depth is well-established for characterization of organic semiconductors and their properties. Grazing incidence small and wide angle scattering (GISAXS, GIWAXS) as well as grazing incidence X-ray diffraction (GIXD) are widely used methods for functional layers characterization of organic electronics device prototypes [19]. Benefits of the technique already have been demonstrated in various studies of the material structure for organic electronics. In situ investigations of organic transistors were performed and reported based on absorption spectroscopy [20, 21], X-ray diffraction experiments [22, 23] or combination of both [4]. A structural stability of 5,5′-bis(naphth-2-yl)-2,2′-bithiophene (NaT_2_)-based OFETs was studied during operando experiment in combination with GIXD technique. A device with 500-μm-long channel was tested by submillimeter X-ray beam. Rather detail analysis of the material structure demonstrated a high structural stability with variation of 1% in range of up to 40 V [24]. Strong contribution to pentacene-based OFETs was demonstrated from another operando experiment combing with X-ray diffraction [25]. In this work, nanometer-sized monolayers system response on external applied voltage was monitored by XRD. The studies demonstrated slight tilt and reorganization of the molecules at grain boundaries, under applied voltage molecules forming highly crystalline and textured crystal domains.

The structural evolution of the polymer: fullerene active layers during the drying process give insight of the microstructure formation linked to the optimization of the solar cells [26]. Moreover, the solution processing and the microstructure formation can be coupled with electrical response of the active layers [27, 28]. Usually scattering X-ray signal is rather weak in solution phase and at the initial stage of the microstructure formation and such studies would be impossible to realize without usage of synchrotron radiation.

Furthermore, the synchrotron beam focused to submicron or smaller size becomes a necessary tool to achieve a sufficiently high spatial resolution and strong enough signal to monitor microstructural changes during the instrument operation. Typical synchrotron beamlines that deliver a nanometer beam are very spatially limited, what requires from the experimental setup to be compact and flexible. During such investigations, one should consider a number of limiting factors, except abovementioned, which are radiation damage, complicity of contacts alignment, small size of the systems. Importance and complicity of operando synchrotron measurements with nanosize beam were shown in [7] where potential of nanoGIXD experiment was used for a study of real-sized organic field effect transistors under applied voltage.

However, there is no routine way for detailed studies of the material behavior under real working conditions. Particular difficulties are related to the small dimensions of the active layers of the modern smart devices and circuits.

In this paper, we present a self-developed setup for in situ studies under applied voltage, which is compact and compatible with different synchrotron beamlines. The voltage in situ setup for Application at Synchrotron beamlines (*VINAS*) is a new tool dedicated for operando studies of the multilayer-based system that supposed to be operated under external voltage conditions. A self-explanatory and easy-to-use setup simplifies the electrical connection and eliminates any blocking of an X-ray beam. *VINAS* can be operated under broad ranges of applied voltages, which make the setup helpful for studies of almost all types of new vertical microelectronic devices. The potential of the setup is demonstrated on operando studies of working prototype OFET device based on DH4T oligothiophene. Results obtained during the reported experiment complete the knowledge of the device stability under externally controlled experimental conditions.

## Methods

### Setup Description

**Fig. 1:**
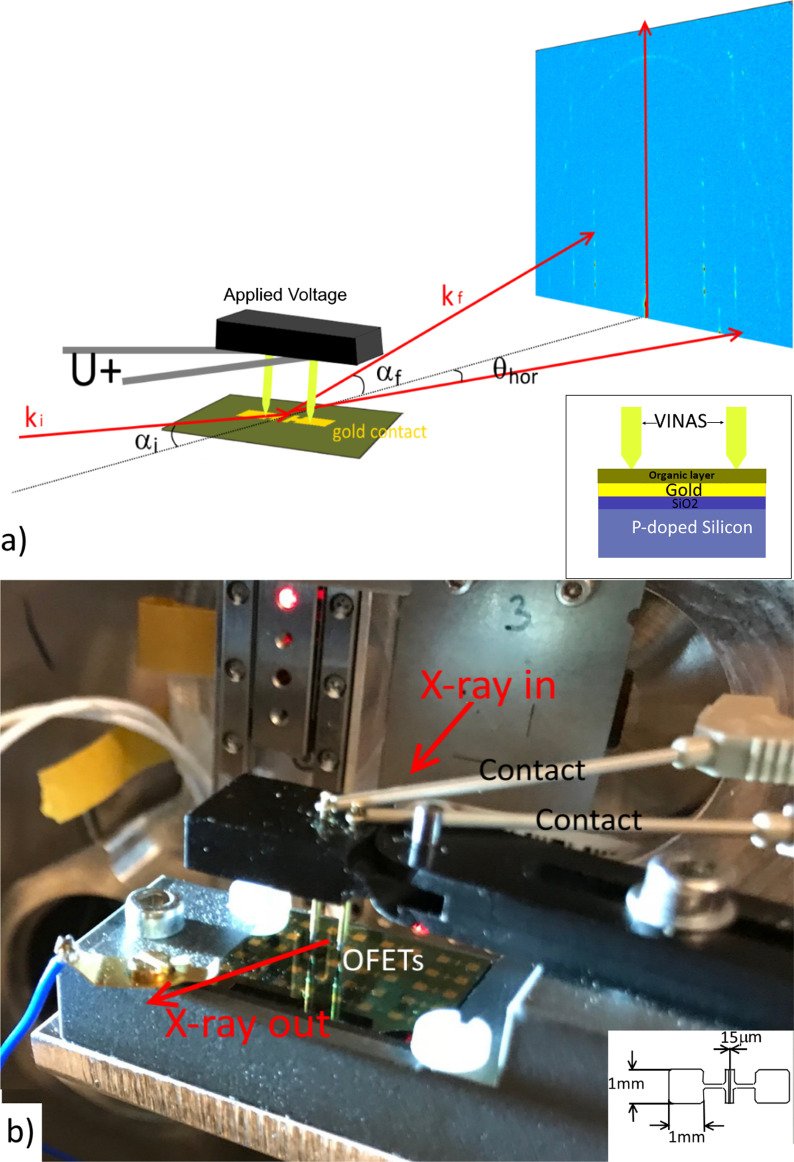
**a** Scheme of the *VINAS* setup with installed sample and marked vertical contacts during in situ measurements; **b** photograph of *VINAS* setup installed at P03 nanofocus endstation (PETRA III, DESY), real setup of reported studies, inset: schematic image of the OFET device

The voltage in situ setup for application at synchrotron beamlines is a compact device compatible with various synchrotron beamlines for in situ studies of a multilayers system with promising electrical properties. The *VINAS setup* is self-developed device designed and constructed by Soft matter physics group (University of Siegen, Germany). The potential of the setup was explored by operando studies of the OFETs devices based on DH4T. Circuit board with the sets of 20 OFET devices was fixed by special plastic screws on the board corners in harmless way. OFETs themselves are multilayer system (see schematical inset in Fig. 1a) consisting of a silicon substrate, followed by a 300-nm-thick silicon oxide layer with thermally evaporated gold electrodes on top of it. Finally, a 100 nm DH4T thin layer was thermally evaporated making bottom source–drain, bottom gate device. For sake of simplification, the gold electrodes are shown uncovered in Fig. 1a. Special probing setup enables the voltage to be applied to any of the selected transistor by vertical 7-mm-long electrodes leaving free space for the incoming and scattered X-ray beam (see Fig. 1). A custom-made setup enables local positioning of all 20 different single-channel OFETs using special gold-sputtered pins with springs to connect to the contact pads of the single-channel OFETs in the bottom contact geometry. For operando studies, we have chosen central OFETs with the channel length of 15 µm and channel width of 1 mm.

### X-ray Characterization

The reported experiment was performed at the Nanofocus Endstation of P03 beamline at PETRA III Synchrotron, DESY (Hamburg, Germany) [29]. The VINAS setup (Fig. 1b) was installed on a hexapod from SmarAct that allows scanning along three orthogonal axes and corresponding angles. Monochromatic synchrotron beam with photon energy of 16.98 keV was focused down to size of 300 × 300 nm^2^ by means of Kirkpatrick–Baez mirrors. The measurements were performed in GIWAXS geometry with the angle of incidence *α*_*i*_ = 0.045°. Such shallow angle resulted in footprint of around 400 um which enables to enhance scattering signal and to measure microstructure along main part of the active channel (with the width of 1 mm). Scattered signal was recorded by CCD type Photonic Science area detector with pixel size of 62 × 62 µm^2^ and dimensions of 2940 × 2940 pixels^2^ installed on the distance of 248.9 mm from the sample position.

### Data Analysis

The data analysis performed with the use of DPDAK software [30] and self-written codes on MATLAB. All peak parameters (as peak position, FWHM, peak area) were obtained through the fitting procedure with Gaussian function.

### Sample Preparation

The OFET testbeds with 20 different devices on a single chip were purchased from Ossila Limited, Sheffield, United Kingdom. The DH4T thin layer was thermally evaporated in a vacuum deposition chamber under high vacuum at 10^−6^ mbar with the evaporation rate of 0.2 Å/s at room temperature.

### Materials Synthesis and Characterization

**Fig. 2:**
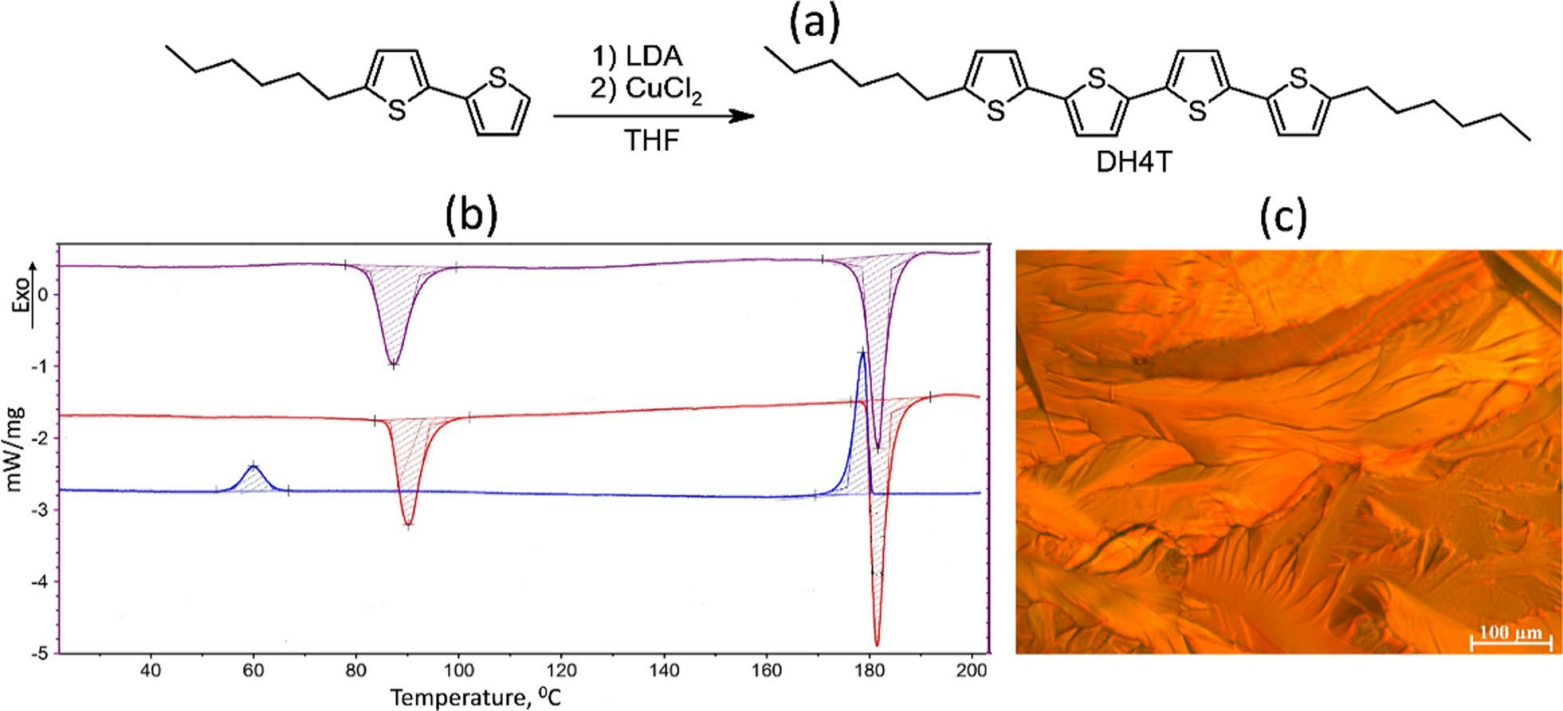
**a** Synthesis of DH4T; **b** heating–cooling–heating DSC cycle of DH4T; **c** polarized-light microscopy image of the DH4T mesophase

D4HT was synthesized through oxidative coupling reaction of lithium derivative of 5′-hexyl-2,2′-bithienyl-5-yl at the presence of CuCl2 (See Fig. 2a), similar to the method described previously [31]. A solution of lithium diisopropylamide (LDA; 1.61 ml of 2 M solution in THF/heptane/ethylbenzene, 3.2 mmol) was added dropwise to the solution of 5-Hexyl-2,2’- bithiophene (0.97 g, 3.9 mmol) in anhydrous THF (15 ml) at − 78 °C under nitrogen. It was stirred for 30 min at 278 °C, and anhydrous powdered CuCl2 (440 mg, 3.3 mmol) was added in one portion to the white-yellowish suspension, upon which the color of the solution changed to a black. The mixture was stirred until it returned to room temperature and then for an additional 60 min at 30 °C. The mixture was poured into 200 ml of water, containing 10 ml of 1 M hydrochloric acid. Then, 300 ml of diethyl ether was added and a yellow solid formed in the ether phase, which was washed three times with 200 ml of water and filtered to give a yellow precipitate, which was washed with diethyl ether and dried under vacuum to give. The crude product was purified by passing through silica gel column (eluent—warm toluene) followed by recrystallization from toluene/hexane mixture to give 500 mg (63%) of yellow crystals. Chemical structure and purity of D4HT were proven by ^1^H NMR spectroscopy and elemental analysis. ^1^H NMR (250 MHz, CDCl3, TMS/ppm): 0.89 (t, 6H, *J* = 6.7 Hz), 1.23–1.45 (overlapped peaks, 12 H), 1.67 (m, 4H), 2.78 (t, 4H, *J* = 7.3 Hz), 6.67 (d, 2H, *J* = 3.7 Hz), 6.96 (d, 2H, *J* = 3.4 Hz), 6.99 (d, 2H, *J* = 3.7 Hz), 7.01 (d, 2H, *J* = 3.7 Hz). Calc.for C28H34S4: C, 67.42; H, 6.87; S, 25.71. Found: C, 67.31; H,6.91; S, 25.66%.

Differential scanning calorimetry (DSC) scans were obtained with PerkinElmer DSC7 system with 10 °C/min heating rate in temperature range of 30–200 °C for all compounds (Fig. 2c). Nitrogen flow of 50 mL/min was used. Optical polarizing microscopy was performed on Axioscop 40 A Pol, Carl Zeiss (Germany) equipped with a heating module.

## Results and Discussion

**Fig. 3:**
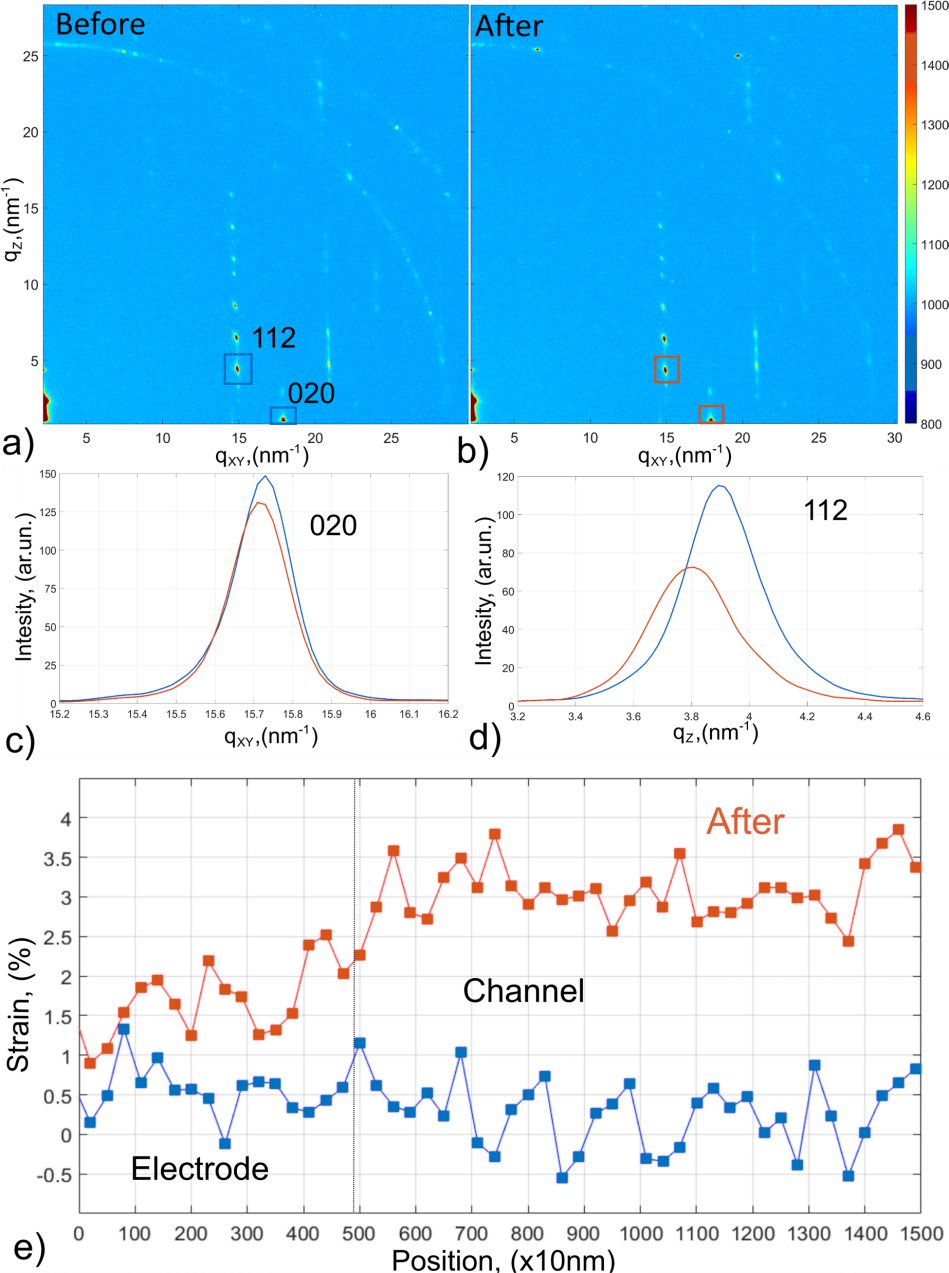
**a** X-ray scattering pattern obtained from the investigated sample before voltage was applied, X-ray peaks selected for further analysis in-plane and out-of-plane structure analysis; **b** scattering pattern after applied voltage **c** in-plane integrated intensity before and after applied voltage; **d** out-of-plane integrated intensity before and after applied voltages; **e** strain evolution along the tested transistor before and after applied voltages

The VINAS setup (Fig. 1) was used for in-operando test of organic transistor prototype based on DH4T being one of the well-known and promising p-type organic semiconducting materials for further application in organic electronics. Before the reported studies, crystallinity of the organic layer was characterized by DSC heating scans. These scans of DH4T exhibit two peaks at 87 °C and 182 °C, corresponding to the first-order transitions (Fig. 2a) with enthalpies of 36 and 63 Jg-1, respectively. As-received the material in a crystalline phase [32], which changes to a smectic mesophase above 87 °C (Fig. 2b) [33]. Sample with a crystalline layer of 100 nm thickness was used as basis for the OFET prototype manufacture. The device was prepared with a length of the transistor active channel of 15 μm. In addition, electrical response of the selected device was obtained proving possible OFETs characteristics of the device. The sample was placed to *VINAS* setup installed at P03 nanofocus endstation of PETRAIII synchrotron. Figure 1a exhibits a scheme of X-ray beam scattering from the sample plate toward detector plane. VINAS vertical contacts were placed on gold electrodes of the transistor, and all system was aligned according the beam position by means of an optical microscope directed along the beam provided by the beamline. Focal plane of the microscope is aligned with the focal plane of KB-mirrors ensuring sample alignment according the focused beam position. Photograph of the experimental setup is shown in Fig. 1b demonstrating a sample positioning and its electrical connection regarding synchrotron beam. An alignment procedure of the sample with already installed vertical contacts to the device electrodes consisted of iterative scanning of height, incident angle and tilt of the sample. Nanobeam is extremely sensitive to any minimal misalignment of the system, so we performed the alignments at two different positions of device at the boarder of the channel and in the middle. All found positions where defined as zeros. Incident angle was chosen *α*_*i*_ = 0.045° smaller than the critical angle of Si substrate at the operated photon energy. Selected incident angle enables to reduce an exposition time and to minimize radiation damage. We scanned along the DH4T transistor channel (Fig. 1b inset) with step of 300 nm (lateral beamsize) and exposure time of 5 s/point—a compromise between a good scattering signal and radiation damage. Radiation damage was measured as integrated intensity signal for prolonged time in the same position of the active channel. These intensity measurements reveal microstructural stability during 3000 s. Prior of operando test, an optimal exposure time was chosen and preliminary lateral scan along the channel was performed. Initial structure of the oligothiophene layer with beamsize resolution was obtained for further comparison. Typical scattering pattern measured at the starting point of the scan at the beginning of transistor channel shown in Fig. 3a, b demonstrates the scattering pattern measured at the same position after in-operando test, when applied voltage was removed. On the both patterns are clearly visible three strong vertical rods of 11L, 02L and 12L families containing number of intense X-ray diffraction peaks and exhibiting highly oriented and well-ordered structure which corresponds to the crystalline phase revealed by DSC. This ordered crystalline phase of DH4T can be described by monoclinic unit cell with the following parameters: *a* = 6.0 Å, *b* = 7.8 Å, *c* = 28.5 Å, and *β* = 93.0° [15]. Further on we concentrated on the selected areas of two vertical truncation rods attributed to reflections family of 11L and 02L for lower and higher *q*_*xy*_ position correspondingly (Fig. 3a, b). Average distance between the peaks in vertical direction for the 11L rod at *q*_*xy*_ = 13.2 nm^−1^ corresponds to 2.9 nm in real space, what is in a good agreement with vertical stacking distances between molecules. No difference in the average peaks distance is observed comparing diffraction patterns before and after the test (Fig. 3a, b). In addition, two ring-like signals at higher *q* values could be observed from the pattern corresponding to Au 111 and 200 reflections, which originated by the gold electrode.

**Fig. 4:**
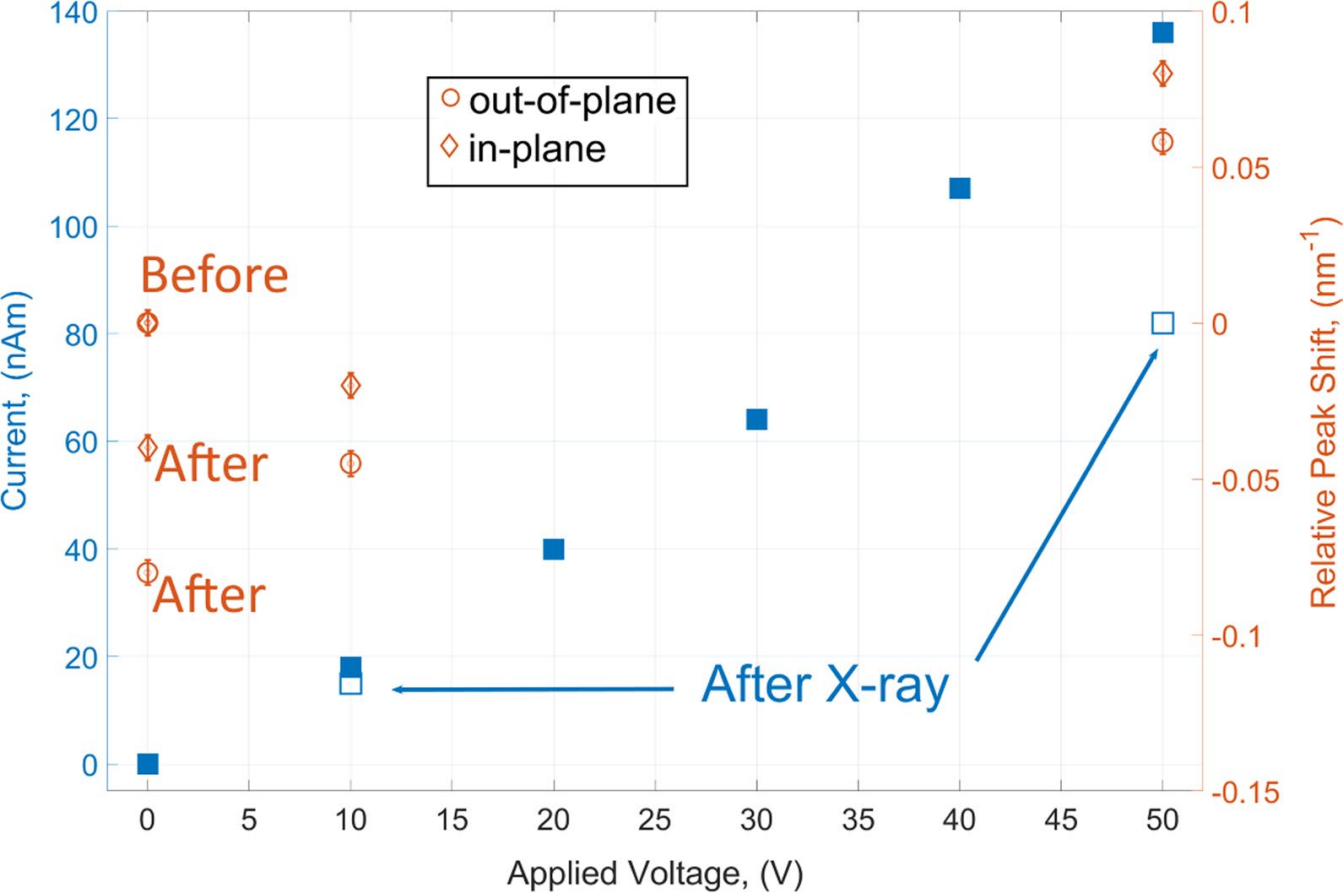
At the blue axis: current response to the applied voltage during (filled squares) and after(empty squares) X-ray measurements; at brown axis: relative shift of the average peak position in in-plane(diamonds) and out-of-plane(circles) directions

We have selected 020 and 112 reflections to characterize structural changes in the in-plane and out-of-plane directions (marked areas in Fig. 3a). We compared changes of scattering signal at the initial stage (before voltage was applied) and after all measurements (when voltage was removed), considering that irreversible changes might be originated by the prototype operation. The peaks shape and position of the selected Bragg reflections were evaluated by fitting integrated intensity curves employing the Gaussian functions. The line profiles for 020 reflection integrated in in-plane direction are shown in Fig. 3c and do not exhibit major changes in both shape and position remain relatively stable along entire experiment. In the out-of-plane direction (integrated 112 reflections Fig. 3d), stronger changes take place with substantial broadening of the peak and expansion of the interplanar distances. Applied voltage originates lattice expansion in the vertical direction and misorientation of the structure shifting positions from initial almost upright-standing molecules. These findings are supported by peak shifts in direction of smaller q and from peak broadening after the prototype exploitation. Overall in-plane microstructure with improved π–π stacking reveals to be more stable than out-of-plane one associated with long axis of small molecules which includes flexible chains. The tendency is clearly demonstrated by the comparison of initial and final measurements in Fig. 3c, d. Interestingly, reorientation of the molecules with various tilt angles depending on applied thermal budget was observed and discussed for DH4T and DH5T oligothiophenes in [15, 22], correspondingly. The lattice expansion originates strain fields that can be evaluated from obtained data with hundreds-of-nanometer resolution. Figure 3e demonstrates spatially resolved strains profiles in out-of-plane direction along the transistor channel with beamsize resolution (300 nm) before and after applied voltages. Here, strains are calculated as a deference of the spacing between molecules (*d* − *d*_0_) divided by the initial spacing (*d*_0_). At the first part (from 0 to 500 nm) corresponding to the film deposited on the gold electrode, the strain is slightly higher than 0 initially. The area from 500 to 1500 nm refers to the conducting channel and is initially strain-free. Further, a remarkable difference between two areas on the gold and in conducting channel is observed. The changes of the oligomeric molecules on the gold electrode are weaker showing strain values increase of about 1%. This is not the case for the stage after operando test in the conducting channel area where remaining vertical lattice strains increase up to 3%. Interestingly, local scale GIXD studies of poly(3-hexylthiophene) OFETs revealed also the most pronounced changes in microstructural order the interfacial regions of the gold electrodes and the conductive polymer channel and when an electric field was applied [7]. In current work, we are reporting on in situ results obtained with the selected DH4T organic material under fixed applied voltages for the demonstration of the setup feasibility. The OFET mobilities for oligothiophenes with the linear alkyl groups including DH4T were found to be in the range from 0.0004 to 0.08 cm^2^ V^−1^ s^−1^ [14, 21]. More detailed in situ structural and electrical characterization of oligothiophenes will be reported elsewhere. Figure 4 on a blue axis demonstrates the prototype electrical response on the applied voltage during and after X-ray measurements. The current increases almost linearly from 13.3 nA at 10 V and reaching 136.2 nA at 50 V. After X-ray scans during 1500 s at the fixed value of 50 V, the current has lowered of about 30%. Afterward, coming back to the 10 V system has recovered and shows almost same level of the current (empty blue squares, Fig. 4). The lattice response of DH4T under applied voltages is shown on brown axis of Fig. 4 in terms of the relative displacements of the peaks positions in reciprocal space in both directions in-plane(diamond) and out-of-plane(circle). When the voltage of 10 V was applied, almost no displacement in the in-plane direction was observed, while at the same time the out-of-plane peak moved in the negative direction for 0.05 nm^−1^. With increasing voltage to 50 V, we observe shifts in the positive direction for both cases—for out-of-plane 0.1 nm^−1^ and 0.09 nm^−1^ for in-plane components in total. After the voltage was completely removed, the in-plane component returned almost reversibly to the initial value—differs only for 0.017 nm^−1^, the out-of-plane directions were more sensitive again and show differences of 0.155 nm^−1^ compared to the previous position and 0.096 nm^−1^ compared to the initial stage. From this, we can conclude that the orientation of the structural blocks—molecules—in-plane direction is more stable than in normal direction to the sample surface, but the stability over all is still better compared to other materials in both directions [7, 22]. There are no significant changes in the position of in-plane peak and overall trends supports higher stability of in-plane components in comparison with out-of-plane ones.

The results of operando tests on semiconductor polymer OFETs with nanosized synchrotron beam and their importance for the material studies were reported earlier [7]. Such experiments are faced to a number of technical challenges related to precise positioning of the electrical connectors and restricted volume for the sample environment. The use of VINAS allows to decrease a time required for alignments and electrodes connections with a sample and to provide a stable setup well suitable for in situ investigations. The VINAS has been successfully operated for another type of oligothiophenes during in situ testing (to be published). Despite the weak scattering signal due to X-ray focusing, good quality materials’ response could be obtained by increasing the exposure time. Stable in-operando conditions during the measurements are ensured by reported self-developed setup. The chosen experimental conditions enable to perform OFETs stability test and monitor material response on the externally applied voltage. The importance of operando stability tests for novel OFETs devices is clearly underlined by the community, and a development of the universal experimental approach demonstrated here is substantial contribution to the field. For the first time, we have demonstrated that the organic material might be characterized in situ in terms of voltage-induced strains with sub-micron resolution. With increasing of interest in low-costs electronics based on π-conjugated small molecules and polymers, the studies of materials functionality will be pushed to acceleration and request of universal setups for investigations of the material performances will grow.

## Conclusions

In summary, the in situ voltage setup for synchrotron studies has a great potential for the investigation of layer systems under applied voltage by the surface sensitive synchrotron techniques. It allows direct correlations between an externally applied voltage and the response of materials of interest. We have shown the advantages of in situ experiment where different voltages were applied to monitor simultaneously the microstructure and the electrical response. In comparison with conjugated polymers, the thiophene-based D4HT OFETs reveal to be stable under applied voltage conditions where DH4T oligomer preserves its crystalline phase without transition to mesophase. These stable crystalline phase reveals rather week anisotropy of structural properties with minor changes of in-plane components in comparison with out-of-plane ones. The possible applications of the setup are much wider than in the example given here. Considering the intensive studies on transistors based on organic π-conjugated molecules and the importance of operando experiments, such in situ instrument is necessary and could be helpful for many scientific applications. Thanks to the compatibility with various synchrotron beamlines and laboratory sources, VINAS allows in situ X-ray investigations in combination with the applied voltage of almost any multilayer systems.

## Data Availability

The datasets used and/or analyzed during the current study are available from the corresponding author on reasonable request.

## Abbreviations

VINAS: Voltage in situ setup for application at synchrotron beamlines
D4HT: Dihexyl-quarterthiophene
OFET: Organic filed-effect transistors
GISAXS/GIWAXS: Grazing incident small/wide angle X-ray scattering
GIXD: Grazing incident X-ray diffraction
DSC: Differential scanning calorimetry
